# Simultaneous production of syngas and carbon nanotubes from CO_2_/CH_4_ mixture over high-performance NiMo/MgO catalyst

**DOI:** 10.1038/s41598-024-66938-6

**Published:** 2024-07-15

**Authors:** Nonthicha Sae-tang, Supanida Saconsint, Atthapon Srifa, Wanida Koo-Amornpattana, Suttichai Assabumrungrat, Choji Fukuhara, Sakhon Ratchahat

**Affiliations:** 1 Department of Chemical Engineering, Faculty of Engineering, Mahidol University, Nakhon Pathom, 73170 Thailand; 2 Center of Excellence in Catalysis and Catalytic Reaction Engineering, Department of Chemical Engineering, Faculty of Engineering, Chulalongkorn University, Bangkok, 10330 Thailand; 3 Department of Applied Chemistry and Biochemical Engineering, Graduate School of Engineering, Shizuoka University, Shizuoka, 432-8561 Japan

**Keywords:** Direct conversion, Biogas, Dry reforming, Methane decomposition, Carbon nanotubes, Syngas, Renewable energy, Chemical engineering, Materials for energy and catalysis

## Abstract

Direct conversion of biogas via the integrative process of dry reforming of methane (DRM) and catalytic methane decomposition (CDM) has received a great attention as a promising green catalytic process for simultaneous production of syngas and carbon nanotubes (CNTs). In this work, the effects of reaction temperature of 700–1100 °C and CH_4_/CO_2_ ratio of biogas were investigated over NiMo/MgO catalyst in a fixed bed reactor under industrial feed condition of pure biogas. The reaction at 700 °C showed a rapid catalyst deactivation within 3 h due to the formation of amorphous carbon on catalyst surface. At higher temperature of 800–900 °C, the catalyst can perform the excellent performance for producing syngas and carbon nanotubes. Interestingly, the smallest diameter and the highest graphitization of CNTs was obtained at high temperature of 1000 °C, while elevating temperature to 1100 °C leads to agglomeration of Ni particles, resulting in a larger size of CNTs. The reaction temperature exhibits optimum at 800 °C, providing the highest CNTs yield with high graphitization, high syngas purity up to 90.04% with H_2_/CO ratio of 1.1, and high biogas conversion (X_CH4_ = 86.44%, X_CO2_ = 95.62%) with stable performance over 3 h. The typical composition biogas (CH_4_/CO_2_ = 1.5) is favorable for the integration process, while the CO_2_ rich biogas caused a larger grain size of catalyst and a formation of molybdenum oxide nanorods (MoO_3_). The long-term stability of NiMo/MgO catalyst at 800 °C showed a stable trend (> 20 h). The experimental findings confirm that NiMo/MgO can perform the excellent activity and high stability at the optimum condition, allowing the process to be more promising for practical applications.

## Introduction

The rapid increase in greenhouse gases (GHGs) paves global warming as one of the most concerning global issues. Dry reforming of methane (DRM), as one of the three alternative methane reactions for methane reforming, has been considered as an efficient solution for contributing to the depletion of the two main components of greenhouse gases (CO_2_ and CH_4_) through converting it into synthesis gas (the mixture of H_2_ and CO) (Eq. (1)) compared with other two cases (Eqs. (2) and (3))^1–9^. Dry reforming of methane (DRM) is a particularly attractive process owing to this process avoids the expensive and intricate gas separation process separation costs results in the overall operation cost to produce high purity syngas being estimated to be 20% lower^10^. It provides a potential pathway for using biogas due to its high content of CH_4_ and CO_2_. Biogas is considered a renewable and sustainable energy strategy^11^ produced via anaerobic digestion or fermentation of any biodegradable organic matter, including municipal solid waste, sewage sludge, agricultural wastes, animal dung, and energy crops of organic waste materials^12^. According to the world biogas production from 2000 to 2014, biogas increased by 357% to 59 billion m^3^ with forecasts that this will continuously rise steadily^13^. In general, biogas consists of the major components are methane (50–70%) and CO_2_ (30–50%). There are various impurities with very low concentrations such as H_2_, O_2_, N_2_, NH_3_, H_2_S, and H_2_O vary depending on the feedstock^14^. Directly converting biogas is regarded as a simple and eco-friendly approach because it is not only the best management approach for the enormous amounts of bio-waste produced domestically^15^, but it is also a potential pathway for converting high levels of methane and carbon dioxide into more value-added products. The dry reforming reaction (DRM) consumed methane and carbon dioxide resulting in the formation of synthesis gas having a ratio of hydrogen to carbon monoxide close to unity^16^, which can be practically used as a raw material in the Fischer–Tropsch industry to produce a variety of other chemicals platform^17–19^. Meanwhile, during the dry reforming reaction, a part of methane in biogas is consumed in a side reaction known as catalytic decomposition of methane (CDM) (Eq. (4)) to produce carbon nanotubes. This occurs is another advantage of processes due to carbon nanotubes (CNTs) being perceived as valuable carbon nanomaterials, as it possesses a unique for example, excellent structural, physical, electrical, and mechanical properties, resulting in a wide range of applications^20–23^. In this work, we focused on the integrative process of dry reforming of methane (DRM) and catalytic decomposition of methane (CDM) to establish a potential process for completely converting biogas into syngas and CNTs. Generally, the obtained CNTs are considered a main product, while the spent catalyst is impurity. In the CNT purification process, the remaining catalyst can be removed by acid leaching, then the high purity CNTs are obtained. The catalysts dissolved as ions, and it might be recovered. A wiser option is to increase a fraction of carbon to be more than 90%. It is referred to commercial grade CNTs, and it can be used as synthesized without any further purification.

1 $$ {\text{DRM:}}\,\,\,{\text{C}}{{\text{H}}_4} + {\text{C}}{{\text{O}}_2} \to 2{{\text{H}}_2} + 2{\text{CO}}\quad \Delta {\text{H}}_{298{\text{K}}}^{\circ} = + 247\;{\text{kJ mol}}$$

2 $$ {\text{SRM:}}\,\,\,{\text{C}}{{\text{H}}_4} + {{\text{H}}_2}{\text{O}} \to {\text{CO}} + 3{{\text{H}}_2}\quad \Delta {\text{H}}_{298{\text{K}}}^{\circ} = + 206\;{\text{kJ mol}}$$

3 $$ {\text{POM:}}\,\,\,{\text{C}}{{\text{H}}_4} + 1/2{{\text{O}}_2} \to {\text{CO}} + 2{{\text{H}}_2}\quad \Delta {\text{H}}_{298{\text{K}}}^{\circ} = - 36\;{\text{kJ mol}}$$

4 $$ {\text{CDM:}}\,\,\,{\text{C}}{{\text{H}}_4} \to {{\text{C}}_{\text{s}}} + 2{{\text{H}}_2}\quad \Delta {\text{H}}_{298{\text{K}}}^{\circ} = + 75.3\;{\text{kJ mol}}$$

An efficient dry reforming catalyst must be thermally stable and yield optimal conversions. It must also be commercially viable so that it can be scaled up for industrial applications at an affordable cost. Catalyst is considered one of the crucial important factors in achieving the conversion rate of methane and carbon dioxide for dry reforming of methane (DRM) and catalytic decomposition of methane (CDM) as well as significant influences on the yield and the morphology of the synthesized CNTs. Noble (Rh, Ru, Pd, Pt, etc.) and Non-noble (Ni, Co, Fe, etc.) metal catalysts are typically carried out over transition, and noble metal-based catalysts. Nobel metal catalysts exhibit preferable a high catalytic activity and stability, anyhow the extremely high price hinders their further development and widespread use^24–27^. Non-noble metal catalysts have thus attracted much attention as low-cost materials and high activity^28^. Among the non-noble metal catalysts, Ni exhibits the most active catalyst for dry reforming of methane (DRM) and catalytic decomposition of methane (CDM) reaction^29–33^. However, the rapid deactivation during reaction due to metal particles encapsulated by carbon and sintering under operation at high temperature is a major drawback of Ni nanoparticles^34–37^. Numerous studies have been conducted to overcome the problem of Ni nanoparticles by modifying the catalyst and adding second metals as promoters^38,39^. Mo is regarded as one of the most studied introduced for methane decomposition due to Mo in the catalysts enhancing the stabilization and dispersion of active Ni particles^40^. Rastegarpanah et al*.*^41^ investigated that the addition of 10% Mo raised the catalytic activity and durability of 55% Ni/MgO catalysts when compared to the unmodified catalyst. Besides the use of a second metal to combine their individual advantages, we previously investigated the effect of introducing third metal on the performance of the catalysts and the properties of the synthesized CNTs compare with bi-metallic catalysts^42^. The studies were carried out over NiMo/MgO, CoNiMo/MgO, FeNiMo/MgO, and CoFeMo/MgO. According to our studies, we observed that the use of bimetallic (NiMo/MgO) catalyst has outstanding catalytic performance. The high yield of CNTs with the best morphology, purity, and textural properties than the use of trimetallic catalysts were obtained. The addition of CO_2_ into the process can maintain the catalyst stability, compared to the decomposition of pure methane or other hydrocarbons. For example, in comparison to the direct decomposition of C_2_H_6_ for carbon nanotube (CNT) production, the presence of CO_2_ significantly improves the catalyst stability^43^. CO_2_ serves dual functions in CNT synthesis. When reacting with C_2_H_6_, it enhances catalyst stability and contributes to approximately 30% of the CNT production yield. The CNTs can be simultaneously produced at high CNT purity. The process can produce the value-added products in both gaseous products and solid products. CNTs can be applied for many applications, such as batteries, supercapacitors, electronics, adsorbent, and reinforced materials^44^. In addition, biogas can be used without any CO_2_ separation. Cao et al.^45^ found that the enhanced CO_2_ adsorption is crucial for coke gasification, with promoted catalysts showing lower coke yields, compared to the pristine material. The morphology of coke impacts the catalyst deactivation. The amorphous coke has minimal effect, while the graphitic carbon, such as carbon nanotubes, significantly impairs the activity by encapsulating active metals. Charisiou et al*.*^46^ reported that the spent Ni/Al samples shows a large coke formation, especially in the less basic alumina-based materials. This indicates a faster methane decomposition and carbon polymerization process, compared to the CO_2_ activation. However, carbon formation and gasification rates show similar on more basic Ni/LaAl catalysts, with coke deposits as nanotubes on Ni particles.

As a continuous work, this work aims to develop the integrative process of dry reforming (DRM) and catalytic decomposition of methane (CDM) to optimize reaction temperature for the utilization of biogas to completely convert into syngas and carbon nanotubes. This experiment was carried out over a NiMo/MgO catalyst using purified biogas at a fixed ratio of CH_4_/CO_2_ (60/40), which is considered a typical composition^47,48^. The raw biogas usually contains approximately 50–70% CH_4_ and 30–50% CO_2_, humidity, and traces of H_2_S, N_2_, H_2_, depending on the sources of feedstock^49,50^. It accepted that the composition consisting of 60% CH_4_ and 40% CO_2_ represents a typical ratio of CO_2_/CH_4_ in biogas^50^. In addition, the effects of varying ratio of CH_4_/CO_2_ on growth and structure of carbon nanotubes are investigated. The long-term stability under optimization of reaction temperature and the ratio of CH_4_/CO_2_ was performed. The obtained CNTs are thoroughly characterized by various techniques.

## Experimental

### Catalyst preparation

The bimetallic NiMo catalyst supported on MgO was synthesized by the wetness impregnation of two metal precursors: Ni(NO_3_)_2_·6H_2_O and (NH_4_)_6_Mo_7_O_24_·4H_2_O (Sigma Aldrich) with mass ratio of 1:1. The total metal loading of Ni and Mo was set at 30 wt%. Briefly, the calculated metal precursor was dissolved in distilled water until formed a completely clear solution. Further, the solution was dropped into MgO nano-powder (98%, Panreac AppliChem) and stirred at room temperature. After a homogeneous slurry formed, the mixture was evaporated on a hotplate at 80 °C until dried catalyst powder was obtained. The resultant powder was then calcined in a muffle furnace at 500 °C for 3 h with a ramp rate of 10 °C/min.

### Characterization

The crystallinity and crystalline phases of both reduced and spent catalysts were examined by X-ray diffraction (XRD, Bruker, D2 Phaser). The XRD patterns were recorded with diffraction angles 2θ ranging from 10° to 80°. The elemental component of reduced catalysts was determined by X-ray fluorescence (XRF, S8 TIGER, Series 2). The morphology of synthesized CNTs was observed by field-emission scanning electron microscope (FE-SEM, JEOL, JSM-7610F). The internal structure of synthesized CNTs was characterized by field-emission transmission electron microscope (FE-TEM, JEOL, JEM-3100, Japan) at acceleration voltage of 300 kV. The number of walls and interlayer spacing are analyzed from TEM images using ImageJ software. The graphitization of synthesized CNTs were evaluated using Raman spectrophotometer (PerkinElmer® SpectrumTM GX). The purity of the synthesized CNTs analyzed by thermogravimetric analysis (TGA, Mettler Toledo, TGA/DSC1). The sample was heated from room temperature to 800 °C, at heating rate of 10 °C/min under O_2_ flow of 100 ml/min. The textural properties of the synthesized CNTs was analyzed by N_2_ sorption measurement (Micromeritics, TriStar II 3020). The sample was degassed under N_2_ flow at 150 °C for 4 h. The specific surface area was calculated using Brunauer–Emmett–Teller (BET) method, whereas the pore size distribution was estimated according to the Barrete–Joynere–Halenda (BJH) using the desorption isotherm data.

### Catalytic evaluation

**Figure 1:**
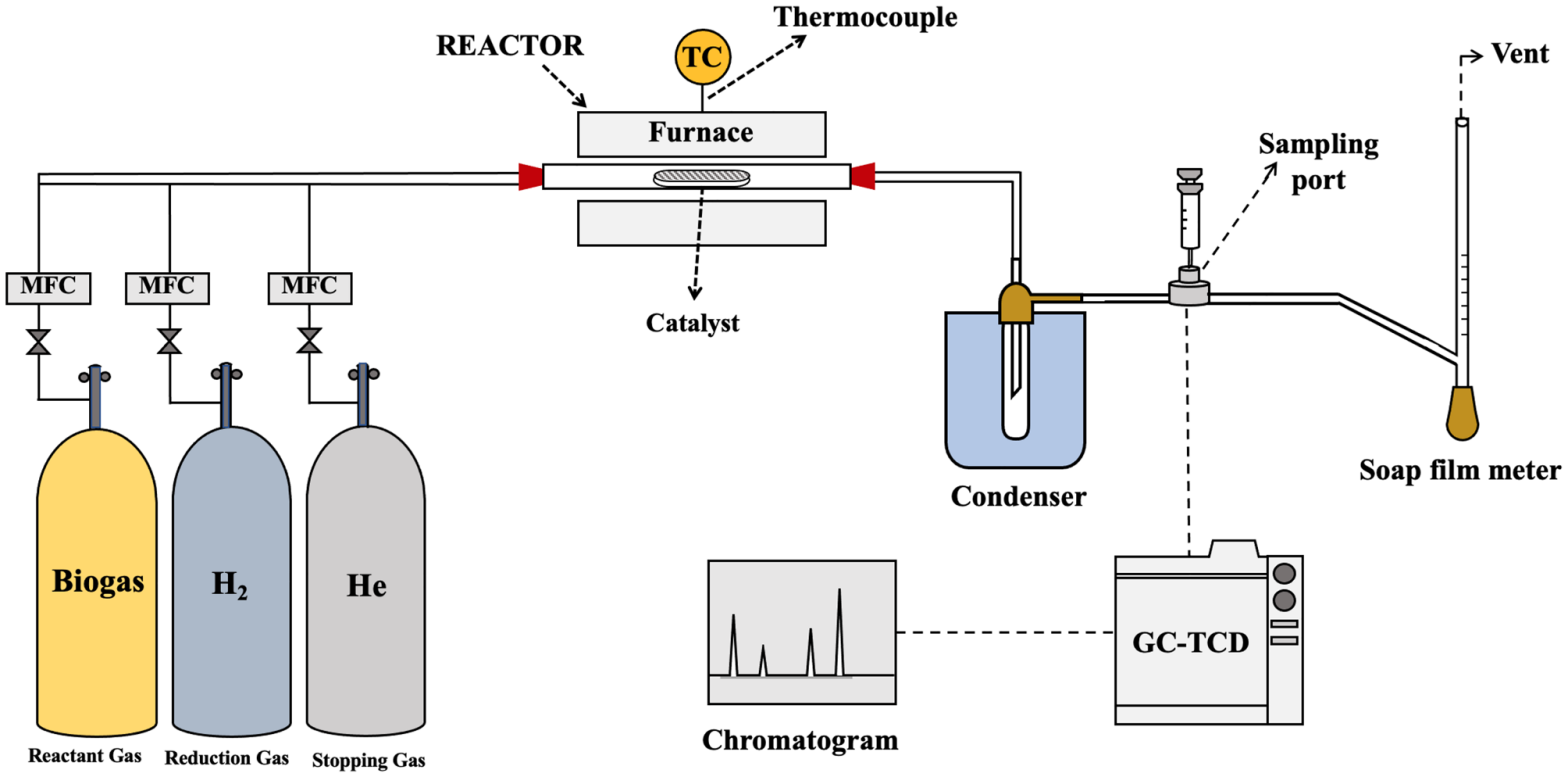
A schematic diagram of experimental setup for production of syngas and carbon nanotubes.

All catalytic performance tests were conducted under atmospheric pressure in the horizontal tubular quartz (2.53 cm inner diameter and 110 cm long) heated by tube furnace, as schematically shown in Fig. 1. A quartz boat (dimension W/H/L: 2/3/10 cm) containing a certain amount of catalyst power was located at the center of the reactor. The catalyst power occupies only the half lower space of cross-sectional area of reactor tube. The space is leaving for CNT growth, avoiding plugging the reactor tube. However, the reaction gases thoroughly diffuse into the sublayer and the bottom bed of the catalyst. This packing of catalyst is a common practice for CNTs production in a fixed bed reactor. The catalyst was first activated by H_2_ reduction process, heated from room temperature to 1000 °C with a ramp of 10 °C/min under H_2_ flow of 75 ml/min. Then, the reactor was set to the designate reaction temperatures, and the model biogas (60% CH_4_ and 40% CO_2,_ CH_4_/CO_2_ = 1.5/1) was introduced to the reactor at 400 ml/min across 0.5 g of NiMo/MgO, corresponding to GHSV = 48,000 ml/g-h. The reaction time was set for 3 h, and during the reaction, the composition of effluent gases was analyzed at regular intervals (10 min) by gas chromatography (TCD, Shimadzu, GC-2014). Finally, after ending the reaction, helium gas was turned into the reactor, while the reactor was cooled down to room temperature. The carbon product together with spent catalyst was collected and kept for further characterization.

This work investigates the effects of reaction temperature, CH_4_/CO_2_ ratio, and catalyst durability. The reaction temperature was studied at 700, 800, 900, and 1000 °C, while CH_4_/CO_2_ ratio was studied at 1.5/1, 1/1, and 0.5/1 at 800 °C. The catalyst durability was tested with the condition: CH_4_/CO_2_ 1.5/1, GHSV = 48,000 ml/g-h at 800 °C for 20 h. However, the initial weight of catalyst was reduced to 0.25 g with a biogas flow of 200 ml/min. The catalytic performance of catalyst was evaluated by conversions of reactants and product yields (syngas and CNTs). The methane conversion (X_CH4_) and carbon dioxide conversion (X_CO2_), and syngas purity were calculated using Eqs. (5)–(7).

5 $${X}_{{\text{CH}}_{4}} (\%) = \frac{{{\text{[CH}}_{4}]}_{\text{in}}\times {\text{F}}_{\text{in }}- {{\text{[CH}}_{4}]}_{\text{out}}\times {\text{F}}_{\text{in}}}{[{{\text{CH}}_{4}]}_{\text{in}}\times {\text{F}}_{\text{in}}} \times 100$$

6 $${X}_{{\text{CO}}_{2}} (\%) = \frac{{{\text{[CO}}_{2}]}_{\text{in}}\times {\text{F}}_{\text{in }}- {{\text{[CO}}_{2}]}_{\text{out}}\times {\text{F}}_{\text{in}}}{[{{\text{CO}}_{2}]}_{\text{in}}\times {\text{F}}_{\text{in}}}\times 100$$

7 $$\text{Syngas purity }(\%) = \frac{({[{\text{H}}_{2}]}_{\text{out}}+{\text{[CO]}}_{\text{out}})\times{\text{F}}_{\text{out}}}{([{\text{CH}}_{4}]_{\text{out}}+{[{\text{CO}}_{2}]}_{\text{ out}}+{[{\text{H}}_{2}]}_{\text{out}}+{\text{[CO]}}_{\text{ out}})\times {\text{F}}_{\text{out}}}\times {100}$$

8 $${{F}}_{\text{i}}\, (\text{ml/min}) = {[{i}]}_{\text{out}}\times{{F}}_{\text{out}}$$

where, [*i*]_in_ and [*i*]_out_ represents the concentration of reactant gas (CH_4_, CO_2_) or product (CO, H_2_) flowing into and out of the reactor. *F*_*in*_ and *F*_*out*_ refer to the total volumetric gas flow rate of the inlet and outlet from the reactor (ml/min). *F*_*i*_ represents the flow rate of each gas species.

In this work, we present CNT productivity in two ways: The CNT productivity was evaluated based on the CNT gram yield and percent carbon yield using Eqs. (9, 10). The gram yield represents the productivity with respect to catalyst, while percent carbon yield indicates the CNT productivity with respect to carbon source. CNT purity was calculated by Eq. (11). The gas hourly velocity (GHSV) was calculated based on Eq. 12.

9 $$\text{CNT gram yield }(\text{gProduct}/\text{gCat h}) = \frac{{{m}}_{{ product}}}{{{m}}_{{catalyst}} \, \times{time}}$$

10 $$\text{Percent carbon yield of CNTs }(\%) = \frac{{{m}}_{{ product}} -{{m }}_{{catalyst}}}{{{m }}_{{catalyst}}}\times 100$$

11 $$\text{CNT purity }(\%) = \frac{{{m}}_{{ product}} - \, {{m }}_{{catalyst}}}{{{m}}_{{product}}}\times 100$$

12 $$\text{GHSV }(\text{ml}/\text{gCat}-\text{h}) = \frac{{F}_{ reactant}}{{{m}}_{{catalyst}} \, }\times 60$$

where *F*_*reactant*_*, m*_*catalyst*_, and *m*_*product*_ represent the volumetric flow rate of reactant gas, the weight of the reduced catalyst, and spent catalyst including carbon formation, respectively.

## Results and discussion

### Characteristics of NiMo/MgO catalyst

The physicochemical properties of as-prepared NiMo/MgO catalyst including elemental composition, crystallinity, and reducibility were analyzed. Table 1 shows the elemental composition of reduced NiMo/MgO catalyst determined by X-ray fluorescence (XRF) and Inductively Coupled plasma (ICP-OES)^42^. It was observed that the total metal loading is 31.8–31.9 wt%, while the mass ratio of Ni to Mo is 0.97–1.07. The results are consistent with the predetermined total metal loading of 30 wt%, and the mass ratio of 1/1, confirming the accurate procedure of catalyst preparation.

**Table 1:** Elemental composition of fresh reduced NiMo/MgO catalyst analyzed by XRF and ICP.

Method	Ni (wt%)	Mo (wt%)	MgO wt%)	NiMo (wt%)	Ni/Mo
XRF	16.5	15.3	68.1	31.8	1.07
ICP	15.7	16.2	68.1	31.9	0.97

**Figure 2:**
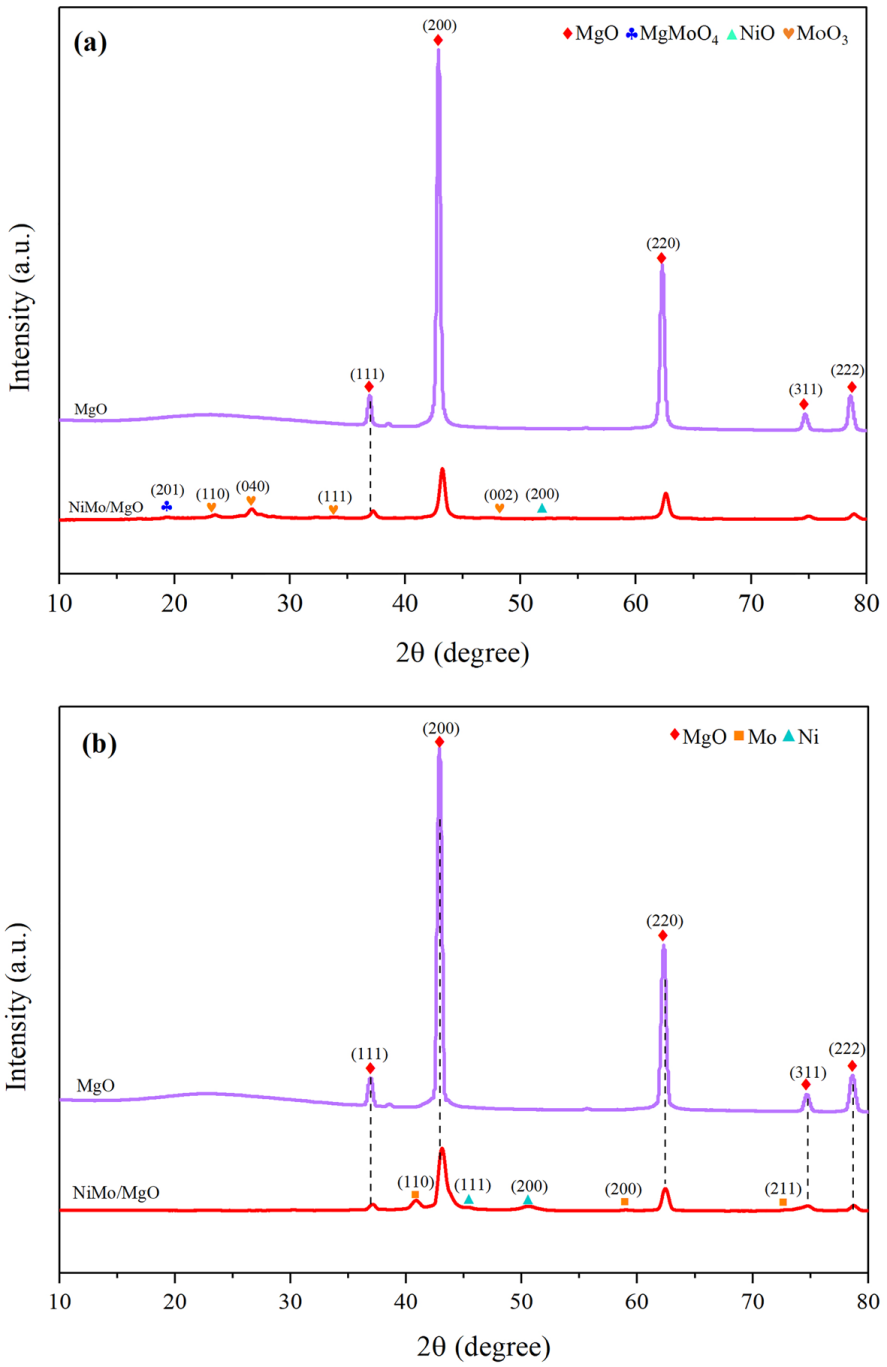
XRD patterns of NiMo/MgO catalysts: (**a**) calcined and (**b**) reduced catalyst.

Crystalline phases of fresh catalysts after being treated in the calcination and the reduction processes were identified by X-ray diffraction (XRD), and the results are depicted in Fig. 2. It was found that the existences of predominant peaks at 2θ = 37, 43, 62, 75, and 78°, which assigned to the (111), (200), (220), (311), and (222) crystal planes of MgO (JCPDS #45-0946) were detected in both calcined and reduced catalysts. In Fig. 2a, the calcination of Mo in the air atmosphere at 500 °C led to the formation of the molybdenum oxide phase (MoO_3_, JCPDS #05-0508), generating the broad reflection peaks (2θ = 23°, 27°, 34°, and 48°). Besides, MgMoO_4_ phase at 2θ = 18° (JCPDS #72-2153) formed by the interaction between MoO_3_ and MgO at temperatures above 400 °C^51^, also appeared with low crystallinity. On the other hand, the characteristic peak of NiO phase (JCPDS #47-1049) was almost absent or undetectable. The reason is probably owing to NiO being well dispersed, corresponding to the introduction of Mo in the catalyst favors the dispersion of Ni^52^. In addition, the absence of Ni^2+^ in Fig. 2b confirmed the dispersion of the Ni–MgO solid solution, causing the peak at 43° to shift to a lower 2θ value which is consistent with the XRD pattern from a previous study^53^. The XRD patterns of reduced catalysts shown in Fig. 2b, it shows no apparent patterns of their metal oxides, indicating that they had been converted into metallic Ni and Mo phases throughout the H_2_ pretreatment process. After reducing the catalyst at 1000 °C, we observed no evidence of MgO sintering. This is likely because MgO nano-powder has closely packed ions, resulting in a high sintering temperature^54^. In addition, Saconsint et al*.* reported that NiMo can stabilize the crystallite size of MgO at 18.1 nm during the reduction process, while the reduction of MgO showed a larger crystallite size of 24.1 nm^42^.

**Figure 3:**
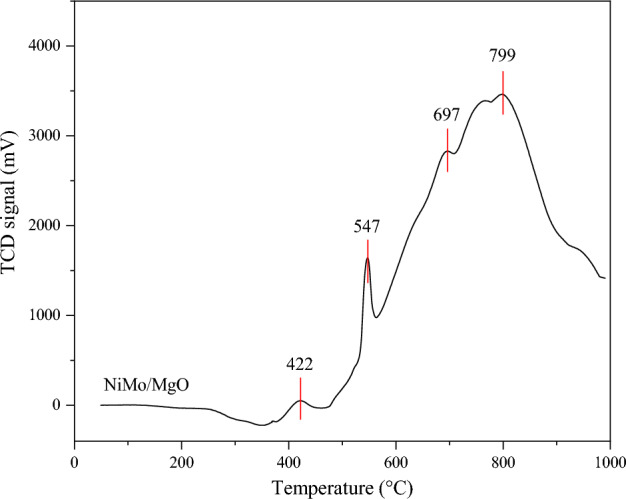
H_2_-TPR profile of NiMo/MgO catalysts.

Figure 3 shows the H_2_-TPR profile of NiMo/MgO catalysts. The fresh catalyst was calcined at 500 °C for 3 h. It was found that the reducibility of catalyst exhibits a broad region between 400 and 1000 °C with four distinct peaks at 422 °C, 547 °C, 697 °C, and 799 °C. The small sharp peak at 422 °C indicates the presence of reducible Ni^2+^ with moderate and mild strength interaction to the support. The second sharp peak at located at 547 °C is ascribed to the reduction of NiO species with strong interaction the support^55^. At 600–1000 °C, the two peaks at 697 and 799 °C, indicating (I) the reduction of Mo^6+^ to Mo^4+^ and the first reduction step of MgMoO_4_, and (II) the second reduction step of MgMoO_4_ and the reduction of Mo^6+^ to metallic Mo^0^. Therefore, the catalyst was intentionally reduced at relatively high temperature of 1000 °C, according to the H_2_-TPR results for the complete reduction.

### Effect of reaction temperature on NiMo/MgO catalytic performance

**Figure 4:**
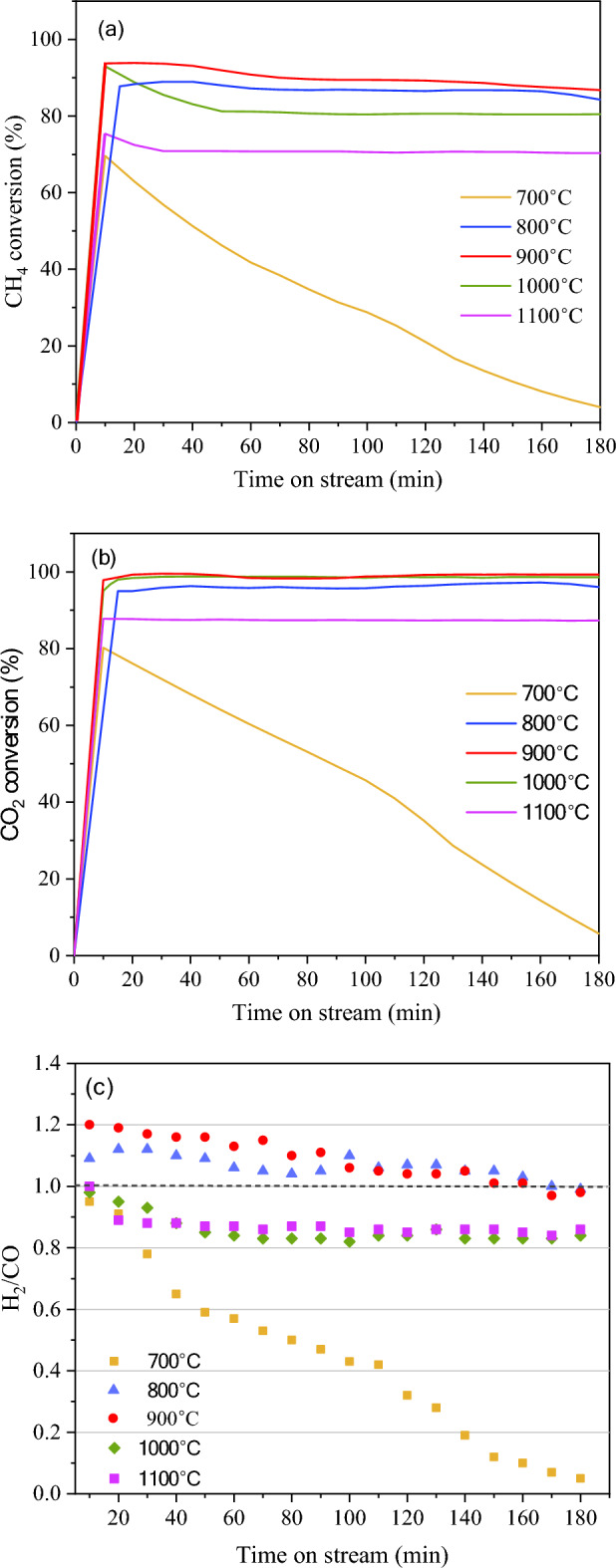
Catalytic activity expressed as (**a**) CH_4_ conversion, (**b**) CO_2_ conversion, and (**c**) H_2_/CO ratio as a function of time on stream over NiMo/MgO catalyst at temperature range of 700–1100 °C, 48,000 mL/g-h, 3 h.

Figure 4 presents the test results of catalytic performance of NiMo/MgO catalyst expressed by CH_4_ conversion, CO_2_ conversion, and H_2_/CO ratio as a function of time on stream (TOS) at different reaction temperatures of 700, 800, 900, 1000, and 1100 °C, while the summary of syngas and CNT products is provided in Table 2. The effects of reaction temperature show that as the temperature elevated from 700 to 900 °C, conversion of both CH_4_ and CO_2_ significantly increased (Fig. 4a,b). Basically, high temperature accelerates the conversion rate of endothermic reactions: dry reforming ($\Delta{\text{H}}_{\text{298K}}^{\circ}= +247 \text{ kJ mol}$) and methane cracking ($\Delta{\text{H}}_{\text{298K}}^{\circ}= +75.3 \text{ kJ mol}$). Further increase in temperature from 900 to 1100 °C, however, would slow down the conversion rates. This result implies that catalyst deactivation appeared at high temperature. High temperature induces migration of Ni-Mo particles, resulting in agglomeration and metal sintering. Notably, more severe catalyst deactivation was observed in low temperature side at 700 °C. The conversion rates rapidly decreased from approximately 60–80% to nearly zero within 3 h TOS. In contrast to high temperature side, the catalyst suffered from carbon deposition at lower temperature. It is apparent that carbon deposit on the spent catalyst at 700 °C, exhibits unique characteristic: low graphitic ratio (Ig/Id = 0.76) and higher deposition temperature (~ 700 °C) (see Fig. 7 and Fig. 8). Even though the amount of deposit carbon only 17.1% compared to other reaction temperatures (84.0, 83.7, 54.9% for 800, 900, 1000 °C), it shows great negative impact on catalyst performance.

**Table 2:** Summary of CNTs yield, CNTs purity, H_2_/CO ratio, and syngas purity after the reaction in the range of 700–1100 °C, 48,000 mL/g-h, 3 h.

Condition	Gram Yield (gProduct/gCat-h)	Carbon Yield (%)^a^	Carbon content (%)^b^	CNT purity (%)^c^	H_2_/CO (–)^d^	Syngas purity (%)
700 °C	0.38	0.2	12.3	19.2	0.4	35.3
800 °C	2.60	11.8	87.2	84.0	1.1	90.0
900 °C	2.50	11.3	86.7	83.7	1.1	93.9
1000 °C	0.75	2.1	55.6	54.9	0.9	88.6
1100 °C	0.50	0.9	33.3	37.1	0.9	77.4
Bayer®	n.d	n.d	–	98.4	–	**–**

^a^Calculation based on carbon content in methane only.

^b^Calculation based on weight gain of spent catalyst.

^c^Analysis by TGA.

^d^Average ratio over 3 h TOS.

Herein, considering the gaseous and solid products of reaction, behaviors of changing syngas and CNT yields (Table 2) are consistent well with the conversion rates as discussed above. Specifically, the temperature elevated (700–900 °C) enhanced overall production yields, while the excessive high temperature (900–1100 °C) reduced the rates of product formation. As a result, yields of syngas and CNTs were maximized at temperatures of 800–900 °C.

It should be noted that CNT yield is highly sensitive to the reaction temperature. The highest CNT yield of 2.60 gProduct/gCat-h was achieved at 800 °C. However, the yield of CNTs was significantly decreased, as the reaction temperature was further increased above 900 °C. At such high temperatures, the dissolving rate of carbon species on catalyst surface would be higher than the rates of diffusion and precipitation, resulting in the accumulation of carbon atoms on the catalyst surface and loss in its activity^56^. Moreover, the high temperature would accelerate the sintering of catalyst particles, leading to loss of activity. As a result, it was concluded that the optimum temperature for the synthesis of CNTs was carried out at 800 °C.

Considering the gaseous products, it was found that the high temperatures (900–1100 °C) would result in a decrease in both syngas yield (Table 2) and H_2_/CO ratio (Fig. 4c). The former was caused by the catalyst deactivation, while the latter was due to, at high temperature, dry reforming (CO_2_ + CH_4_—> 2CO + 2H_2_) was pronounced more than methane cracking. Additionally, the high temperature would promote endothermic reverse water gas shift reaction (Eq. (13)), in which the H_2_ is consumed to produce CO and H_2_O^57^.

13 $${\text{CO}}_{2}+{ \, {\text{H}}}_{2} \, \to{ \, {\text{H}}}_{2}{\text{O}} \, + \, \text{CO } \, \, \Delta{\text{H}}_{\text{298K}}^{\circ} \, = + \text{41.2 kJ/mol}$$

Among the investigated temperatures, it can be concluded that the reaction at 800 °C could be appropriate temperature for simultaneously producing syngas and CNTs at high yields, as well as maintaining best catalyst stability over 3 h TOS. Although the temperature at 900 °C could provide the highest conversions of CH_4_ and CO_2_ at 90.10% and 98.97% and the highest syngas purity of 93.9%, the conversion rates gradually decreased after 40 min TOS, indicating catalyst deactivation.

### Effect of reaction temperature on CNT characteristics

**Figure 5:**
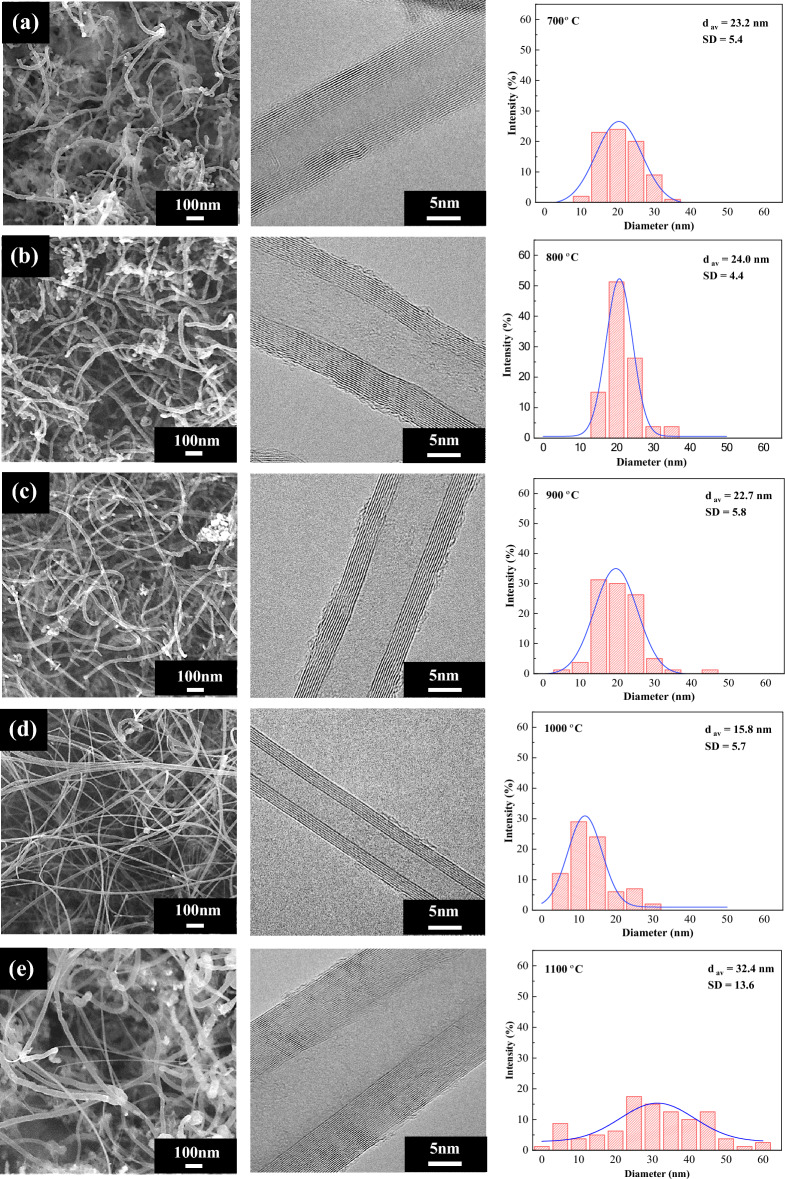
FE-SEM (× 50 k, 5 kV), HR-TEM (× 600 k, 300 kV) micrographs, and diameter distribution of synthesized CNTs over NiMo/MgO catalyst at different temperatures: (**a**) 700 °C, (**b**) 800 °C, (**c**) 900 °C, (**d**) 1000 °C, and (**e**) 1100 °C.

The characteristics of CNTs synthesized over NiMo/MgO catalysts at different temperatures, including morphology, internal structure, crystallinity, graphitization, purity, and textural properties were examined by SEM, TEM, XRD, Raman, TGA, and BET analyses. Figure 5 presents SEM images, TEM images, and corresponding diameter distribution of synthesized CNTs over NiMo/MgO catalyst at different temperatures; 700, 800, 900, 1000, and 1100 °C. It is evident that the carbon formed on all catalyst tests consisted of entangle nanofilaments with several graphene layers that are known as multi-walled carbon nanotubes (MWCNTs). Denser MWCNTs were observed over the catalyst surface under temperatures of 800 °C and 900 °C, corresponding to the high resultant yield of carbon received at these operating conditions.

Considering surface morphology and shape of CNTs elucidated by SEM, low temperature side (700–900 °C) produced a distorted/bent nanotube with rough surface, while high temperature side (1000–1100 °C) resulted in more straighter nanotubes with smoother surface. In addition, the length of the tubes become longer, and fewer defects were observed at the outer wall.

Table 3 summarizes the key morphological characteristics of CNTs synthesized over NiMo/MgO catalyst at different temperatures. The diameter distribution, average diameter (d_av_) of the synthesized CNTs measured from several SEM images, as well as the number of wall and the interlayer spacing derived from TEM results. These revealed that CNTs produced at temperatures between 700 and 900 °C show a morphological similarity in terms of primary diameter size (d_av_ = 23–24 nm), diameter size distribution, number of walls, interlayer spacing, and surface morphology. Interestingly, the CNTs produced at 800 °C are not only the highest yield, but also possess the narrowest diameter size distribution with standard deviation of 4.4 nm. Surprisingly, at 1000 °C the CNTs has the smallest diameter of 15.8 nm with larger interlayer spacing of 0.37 nm. Basically, the interlayer spacing is influenced by CNT diameter and the symmetry of layers^58^. At 1100 °C, agglomeration of metal particles began to be obvious, resulting in the larger and wider diameter of CNTs^59^. It is concluded that the reaction at 800 °C shows the optimum temperature for production of CNTs at high yield and uniform diameter.

**Table 3:** Summary of morphology analysis of CNTs synthesized over NiMo/MgO catalyst with different temperatures.

Parameter	Conditions					CNTs (Bayer®)
	700 °C	800 °C	900 °C	1000 °C	1100 °C	
Shape/surface	Distorted/Rough	Distorted/Rough	Straight/Rough	Straight/Smooth	Straight/Smooth	Distorted/Rough
Distribution (nm)	12–38	16–37	9–45	6–34	4–63	10–30
d_av_ (nm)^a^	23.2	24.0	22.7	15.8	32.4	22.0
SD. (nm)^b^	5.4	4.4	5.8	5.7	13.6	3.3
Interlayer (nm)	0.36	0.36	0.36	0.37	0.35	n.d
No. of wall	11–26	11–34	8–11	7–10	17–31	9–24
Type of CNTs	MWCNTs	MWCNTs	MWCNTs	MWCNTs	MWCNTs	MWCNTs

^a^Diameter of CNTs calculated by mode.

^b^Standard deviation of CNTs diameter.

**Figure 6:**
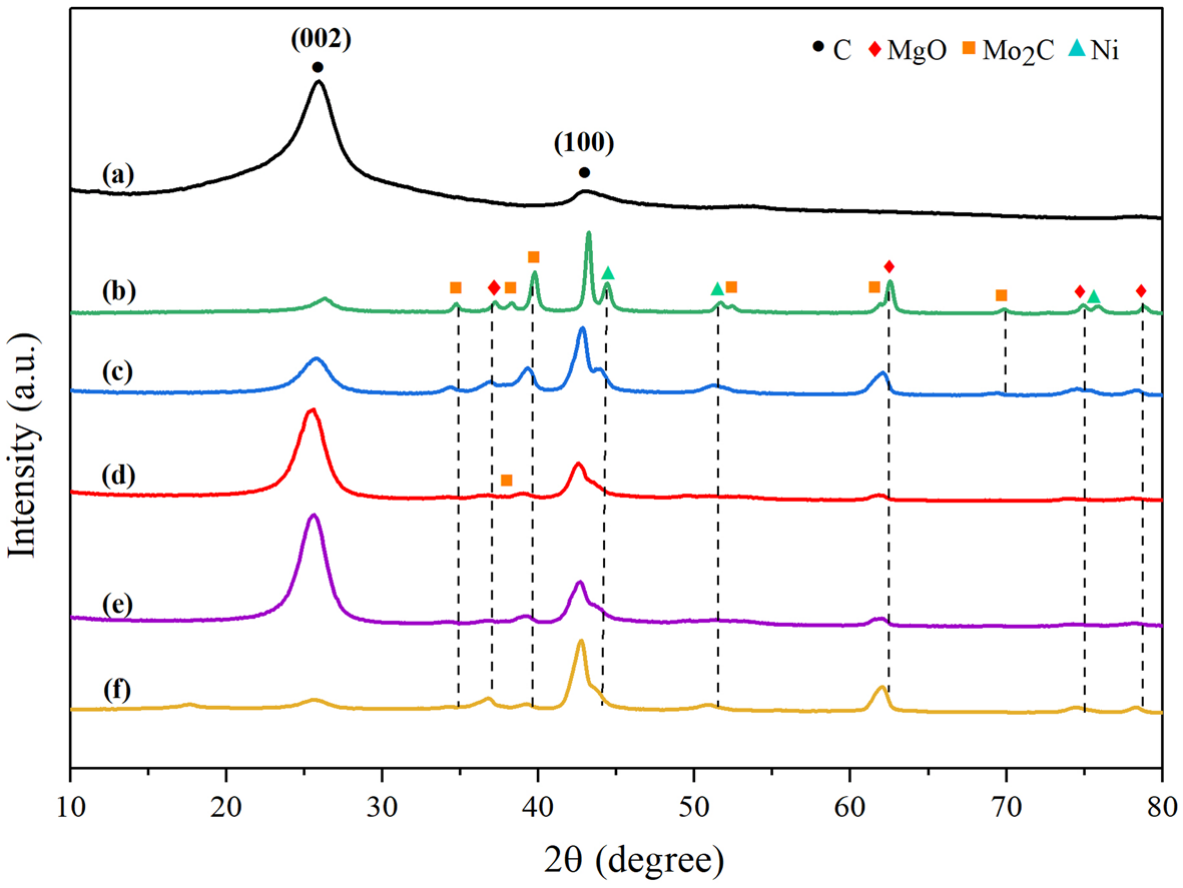
XRD patterns of (**a**) commercial CNTs (Bayer®) and synthesized CNTs over NiMo/MgO catalyst with different operating temperatures: (**b**) 700 °C, (**c**) 800 °C, (**d**) 900 °C, (**e**) 1000 °C, and (**f**) 1100 °C.

Figure 6 presents the XRD patterns of the solid products over NiMo/MgO catalyst after reaction at different temperatures. Compared to the XRD pattern of the fresh reduced catalysts, it is obvious that the reflection peak at 2θ = 26.1° was signified the (002) lattice plane of graphitic carbon materials appeared in all samples. Especially for the diffraction pattern that was performed under temperatures of 800 °C has the highest intensities, which represented the yield of carbon on the spent catalyst, where high catalytic activities for producing carbon nanomaterials display a strong peak. Furthermore, this peak can be used to quantify the level of crystallinity in terms of interplanar spacing as well, *d*_*002*_, which is calculated via Bragg’s equation^60^ and illustrated in Table 4. It was found that all the used catalysts have produced the CNTs with the inter shell spacing value of approximately ~ 0.346 nm, which is even closer to the value of graphite structure (0.335 nm). This shows that flame high-quality crystalline structure of CNTs was grown over the spent catalysts and somewhat improved as the deposition temperature increased. This is also in agreement with the other results obtained in many studies^61–63^. In addition, the diffraction peaks located at 2θ = 42.9° were detected in all samples, where generally attributed to the crystallinity of graphitic carbon as well. However, these peaks overlap with the characteristic peak assigned to MgO support, that is made it difficult to separate peaks. Thus, the relative intensity of this peak does not necessarily indicate the qualitative structural properties of as-grown CNTs.

**Table 4:** XRD data of CNTs synthesized under different deposition temperatures.

Samples	*d*_*002*_^a^ (nm)	*L*_*CNTs*_^b^ (nm)	Mo_2_C (101)	Ni (111)
CNTs_700 °C	0.347	4.96	14.5	n.d
CNTs_800 °C	0.348	4.18	8.3	8.0
CNTs_900 °C	0.349	3.99	12.5	n.d
CNTs_1000 °C	0.346	4.00	10.0	22.6
CNTs_1100 °C	0.340	6.20	22.1	18.0

^a^Interplanar spacing.

^b^Crystallite size for graphitic structure at (002) plane.

**Figure 7:**
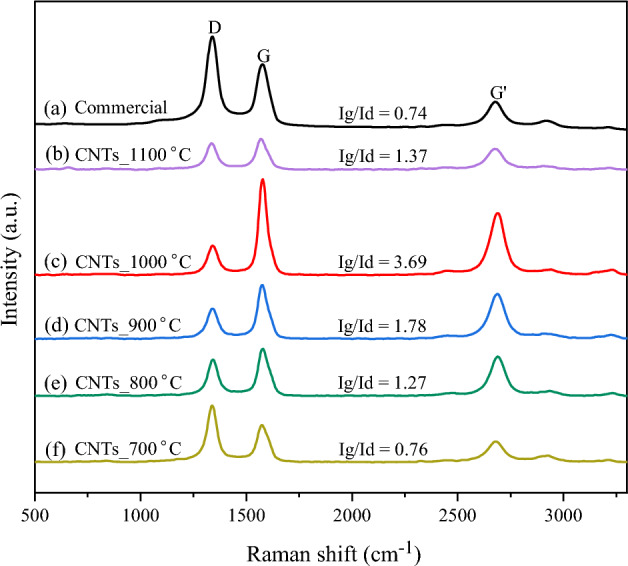
Raman spectra of synthesized CNTs over NiMo/MgO catalyst at difference temperature : (**a**) CNTs_commercial (Bayer®), (**b**) CNTs_1100 °C, (**c**) CNTs_1000 °C, (**d**) CNTs_900 °C, (**e**) CNTs_800 °C, (**f**) CNTs_700 °C.

The graphitization of all synthesized CNTs produced onto NiMo/MgO catalyst at different temperatures was analyzed by Raman spectroscopy as shown in Fig. 7. The G-band (observed at 1500–1600 cm^−1^) is related to the tangential stretching mode of all pairs of *sp*^2^ atoms in both rings and chains which represents the graphitic carbon structure, while D-band (observed at 1300–1400 cm^−1^) is associated with the structural defect and impurity which represents the disordered carbon or amorphous carbon with deposited on the outer surface of carbon nanotube. The quality of carbon nanotube is indicated by the intensity ratio between G-band and D-band (I_g_/I_d_), which higher value (> 1) indicates a good degree of graphitization with fewer defects of carbon nanotube. The results from Raman analysis shows that all synthesized CNTs (I_g_/I_d_ = 0.76–3.69) presented I_g_/I_d_ ratio higher than commercial CNTs (I_g_/I_d_ = 0.74). Apparently, G-band's relative intensity to D-band (I_g_/I_d_) was achieved when the reaction temperature was high as shown in Fig. 7, indicating that a better-graphitized of CNTs was obtained. This occurrence could be ascribed that increases in reaction temperature can increase the solubility of carbon in metal catalysts contributing to the formation of CNTs with greatly graphitized wall structures^59^. This was consistent with the TEM results as shown in Fig. 5 as mentioned above. However, the remarkable decrease in the graphitization at 1100 °C, may be due to the operation under high temperature causing the sintering of metal catalysts leads to lost activity.

Compared to other studies, Charisiou et al*.*^46^ found that both Ni/Al and Ni/LaAl catalysts show that the CNTs contain some defects including amorphous carbon and 16 graphitic layers. However, the high I_G_/I_D_ ratio of 2.86 at 800 °C indicates a high quality of CNTs with a significant graphitic structure. Another research study also supports these findings^4^ suggested that a high I_G_/I_D_ ratio can be achieved at 800 °C despite the presence of some defects. These defects can be reduced by narrowing the D' band. Along with similar reports support these findings^64^ shows that temperature of 750 °C provide high I_G_/I_D_. However, some defects also present in CNTs structure. Pawar et al*.*^65^ proposed that higher temperature could lead to higher carbon nano-structure deposit on Ni/γAl_2_O_3_ catalyst along with increase CH_4_/CO_2_ ratio in comparison.

**Figure 8:**
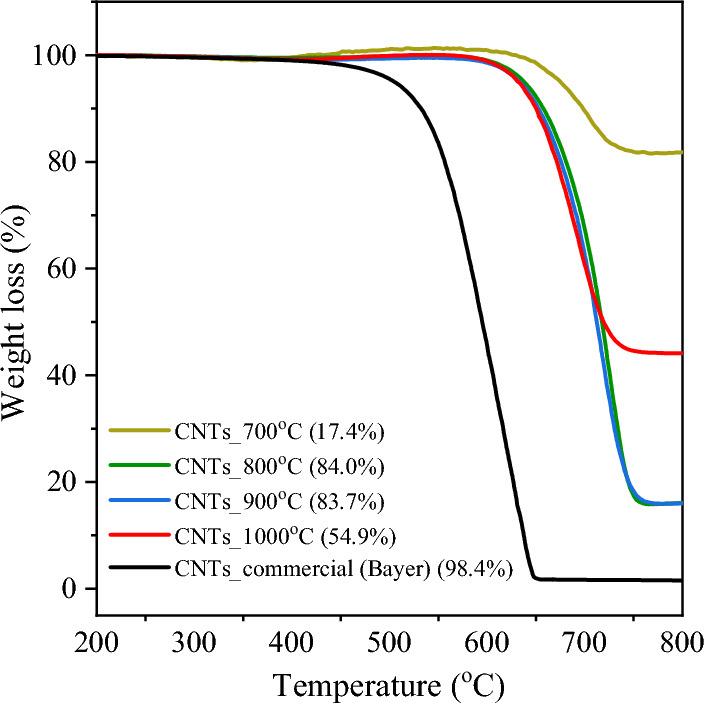
Weight loss curves of CNTs synthesized over NiMo/MgO catalyst with different temperatures compared to commercial CNTs (Bayer®).

Figure 8 illustrates the TGA curves of CNTs products synthesized under varied reaction temperatures. From the TGA analysis, all samples exhibited the same oxidation behavior with a single-step degradation, and no apparent weight loss was observed at temperatures below 400 °C. This may be attributed to their high degree of structural order since the appearance at higher inflection temperature can provide a better crystallinity and thermal stability of MWCNTs^66,67^. In many previous studies, it has also been reported that the percentage of weight loss can refer to the carbon yield in the catalysts that occurs during the oxidation of deposited carbon with oxygen^68^. The purity of CNTs, measured by TGA is presented in Table 2. For temperatures at 800 °C and 900 °C, it is obvious that the small amounts of residue catalyst weight are nearly close to each other, reflecting the improvement of carbon formation with a high purity produced under these reaction conditions, which correlates to the result determined by SEM observation (Fig. 5). Even operating in this region can give better thermal stability with the highest graphitization degree of carbon nanotubes, it seems to show some of the remaining catalysts in the sample, suggesting that it has not able enough to remove the rest of the residual catalysts when compared to the commercial CNTs ones, as displayed in Fig. 8.

**Figure 9:**
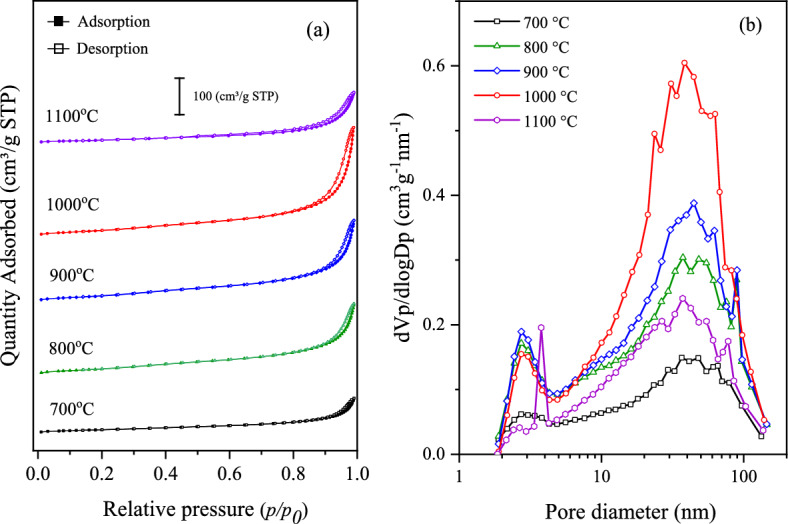
(**a**) N_2_ absorption–desorption isotherms and (**b**) pore size distribution of CNTs synthesized over NiMo/MgO catalyst with different temperatures.

N_2_ sorption measurement is used to assess the textural properties of all synthesized CNTs at different reaction temperatures over NiMo/MgO catalyst, and the results are listed in Table 5 and shown in Fig. 9. It was found to be the specific surface area (S_BET_), mesopore volume (V_meso_), total pore volume (V_total_), and average pore diameter (D_avg_) of synthesized CNTs decreased as the temperature decreased. In Table 5, it can be indicated that all synthesized CNTs are due to the mean pore size of synthesized CNTs being in 11–15 nm which is in the range of mesopore material (2–50 nm). It also confirms by N_2_ absorption–desorption isotherms of all synthesized CNTs shown in Fig. 9a, present type IV isotherm according to IUPAC classification. The hysteresis loops formed by the capillary condensation effect can be classified as H3 hysteretic loop, usually found in solids consisting of aggregates or agglomerates of particles forming slit-shaped pores, with a non-uniform size and/or shape. The pore diameter distributions based on the BJH method as shown in Fig. 9b present the curves, which can be ascribed that all the synthesized CNTs exhibit a bimodal feature which has a pore size with a board distribution ranging from 2 to 140 nm, including a small pore-size fraction (2–4 nm) and a large pore-size fraction (4–150 nm).

**Table 5:** Summary of surface area and porosity of CNTs synthesized over NiMo catalyst with different temperatures.

Conditions	Isotherms	S_BET_ (m^2^/g)	V_meso_ (cm^3^/g)	V_total_ (cm^3^/g)	D_avg_ (nm)
700 °C	Type IV	61.7	0.1564	0.1595	11.5
800 °C	Type IV	127.4	0.3223	0.3273	11.0
900 °C	Type IV	133.0	0.3709	0.3754	11.8
1000 °C	Type IV	133.3	0.4906	0.4958	15.1
1100 °C	Type IV	58.5	0.2299	0.2299	13.8
Bayer®	Type IV	199.8	0.2059	2.4442	48.9

### Effect of biogas composition

**Figure 10:**
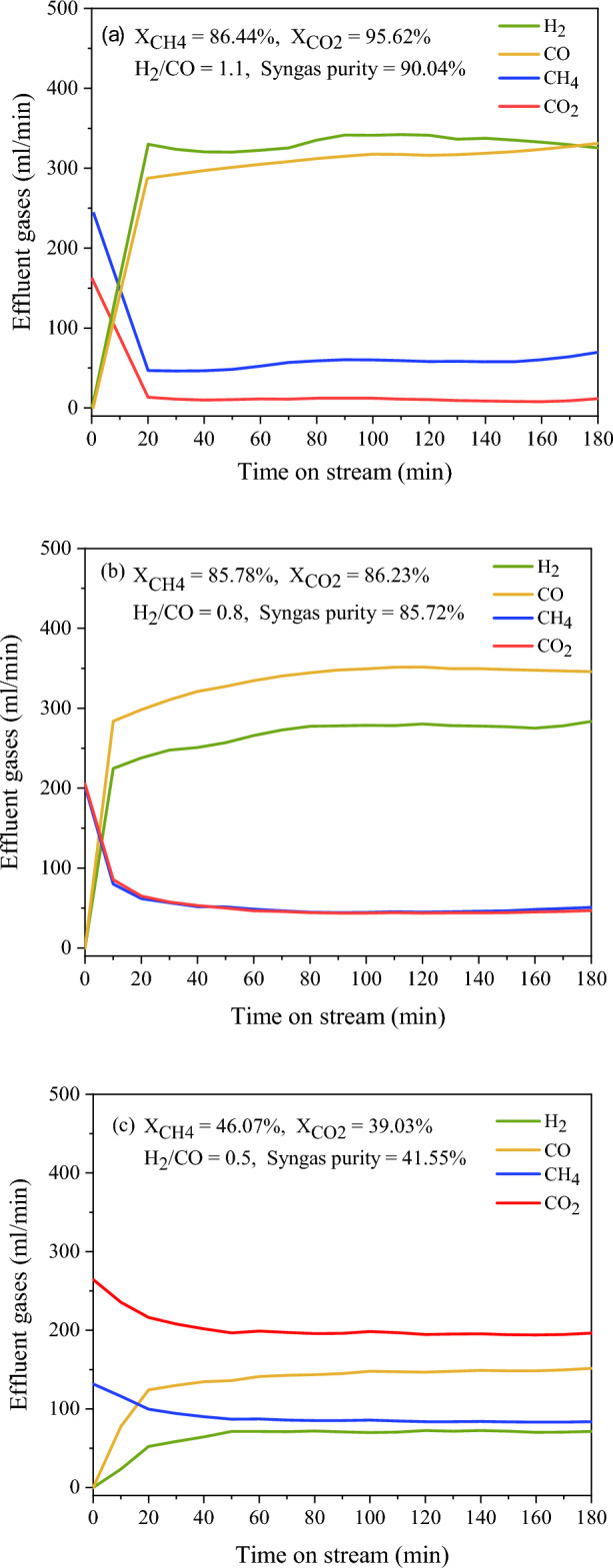
Effluent gases during the reaction over NiMo/MgO catalyst at 800 °C with various feed gases ratio of CH_4_/CO_2_: (**a**) 1.5/1, (**b**) 1/1, (**c**) 0.5/1.

**Figure 11:**
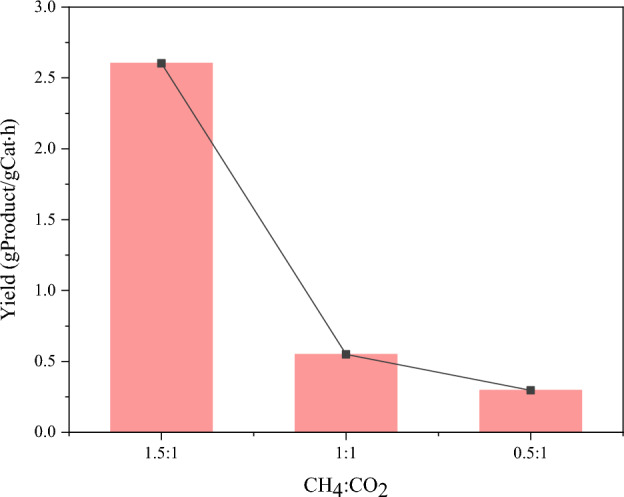
The CNT yield was obtained over NiMo/MgO catalyst at 800 °C with various feed gases ratios of CH_4_/CO_2_.

Generally, the composition of biogas depends on the nature of the material used^69^. The ratio of CH_4_ and CO_2_ in biogas is considered as an important factor in the integration process of DRM and CDM reactions, which may affect the syngas (H_2_ + CO) and CNTs production. In this part, we investigated the effect of the biogas ratio with a variation of CH_4_/CO_2_ in 1.5/1, 1/1, and 0.5/1, which was tested for 3 h. The reaction temperature of 800 °C was selected for carrying out this experiment, based on the previous discussion to identify the optimum condition. The effluent gas composition of outlet stream as a function of times on stream over 3 h. is shown in Fig. 10. In Fig. 10, the biogas using CH_4_/CO_2_ ratio of 1.5/1 achieved higher CH_4_ and CO_2_ conversion of 86.44% and 95.62%, respectively. The existence of an excess of CH_4_ in the feed stream is likely to have produced favorable conditions for the integration of DRM and CDM processes. However, the high concentration of CO_2_ attenuated the CH_4_ and CO_2_ conversion, but it also remains stable over the study time. As a general, DRM reaction is required the CH_4_/CO_2_ ratio of 1/1 based on stoichiometry in Eq. (1) for producing syngas (H_2_ + CO). In this investigation, we found that a CH_4_/CO_2_ ratio of 1.5/1 is more favorable for producing syngas under the study conditions, which contributed to a higher H_2_/CO ratio of 1.1 and syngas purity of 90.04%. The effect of varying biogas feed concentration on the CNTs yield is demonstrated in Fig. 11. One prominent trend is clearly noteworthy that the CNTs yield attained an increase with increasing CH_4_ content in Biogas, implying that a high amount of CH_4_ is sufficient to promote producing CNT through CDM reaction. Therefore, the biogas with CH_4_/CO_2_ ratio of 1.5/1 provided the highest CNTs yield of 2.60 gProduct/gCat-h. As a result of these observations, it was suggested that the biogas used with less CH_4_ content may be unsuitable for simultaneously produced syngas and high value-add carbon nanomaterial.

**Figure 12:**
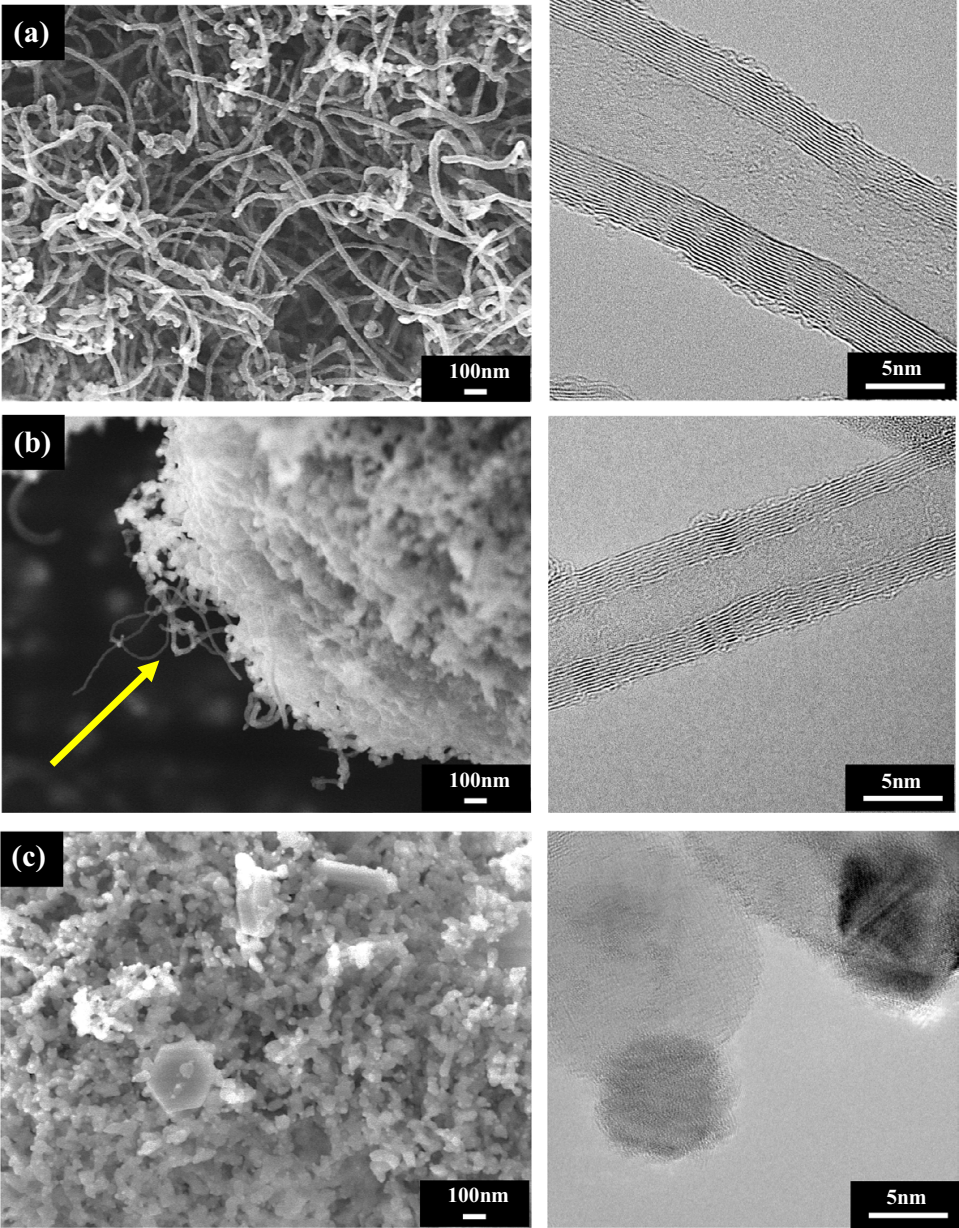
FE-SEM (× 50 k, 5 kV), HR-TEM (× 600 k, 300 kV) micrographs, and diameter size distributions of solid products synthesized over NiMo/MgO catalyst at 800 °C with various feed gas ratio of CH_4_/CO_2_: (**a**) 1.5/1, (**b**) 1/1, and (**c**) 0.5/1.

**Figure 13:**
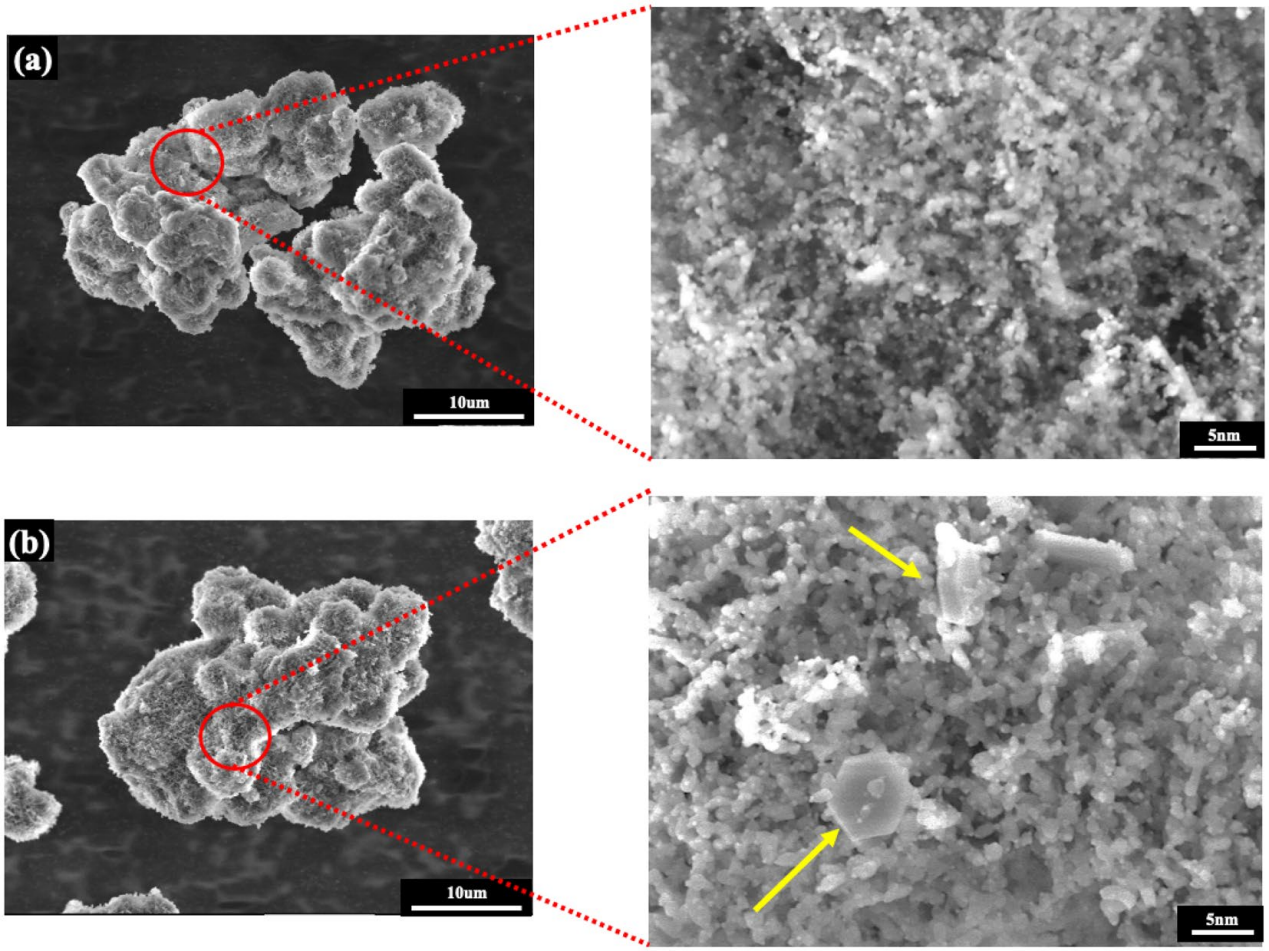
FE-SEM (× 2.5 k, 5 kV) and HR-TEM (× 600 k, 300 kV) micrographs of (**a**) reduced NiMo/MgO catalyst (**b**) solid product after the reaction at 800 °C with feed gas ratio of CH_4_/CO_2_: 0.5/1.

To study the influence of biogas composition, the reaction was carried out at different ratio of CH_4_/CO_2_ from 0.5 to 1.5. The CNTs formed were investigated under FE-SEM and HR-TEM analysis as depicted in Fig. 12. It was clearly observed that the growth of nanotubes are multi-walled CNTs, which were densely presented on the use of the CH_4_/CO_2_ with the highest ratio of 1.5 (Fig. 12a) while the extremely tiny amount of carbon nanotubes was detected only at the edge of the catalyst with a feed ratio of CH_4_/CO_2_ equal to unity and becoming even less or absent when the level of carbon dioxide continuously raised (that is, CH_4_/CO_2_ = 0.5/1) (Fig. 12b,c). According to these findings, it can be implied that increasing the concentration of CO_2_ in the inlet reactant gases decreased the presence of carbon nanomaterial, conversely, inserting more CH_4_ content can promote the growth of CNTs as observed in the yield carbon results. This is probably due to the highly stable of CO_2_ and thus causing it difficult to crack carbon atoms for forming to be solid carbon as a co-product^70^. Furthermore, at the largest amount of feeding CO_2_, it appears obvious that the textural of the catalyst had changed slightly compared to the fresh catalyst before running the reactions as demonstrated in Fig. 13. The CO_2_ oxidation under this circumstance (CH_4_/CO_2_ ratio of 0.5/1) led to a catalyst surface composed of some hexagonal microrods which evidently appeared in the inset SEM image (Fig. 13b) that shows the zoom-in view for the surface morphology of catalyst. This suggests that there is one another form of molybdenum oxide phase (MoO_3_)^71^, which is expected to be an explanation for the absence of CNTs in the sample.

**Figure 14:**
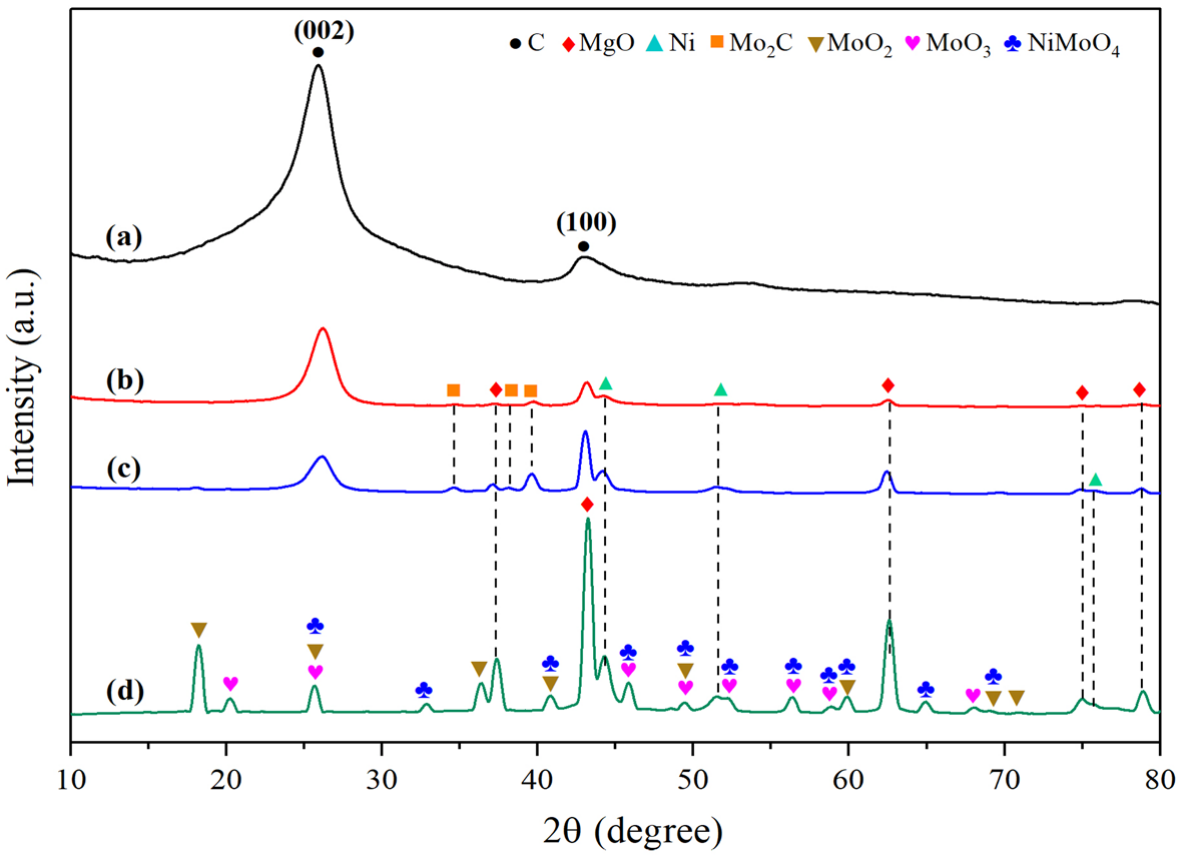
XRD diffraction patterns of (**a**) commercial CNTs (Bayer®) and synthesized CNTs over NiMo/MgO catalyst at 800 °C with various feed gas ratio of CH_4_/CO_2_: (**b**) 1.5/1, (**c**) 1/1, and (**d**) 0.5/1.

From a previous discussion, the arising of the oxide species under varied conditions was further examined using the XRD technique and the results are shown in Fig. 14. Obviously, upon continuing to increase the amount of CO_2_ in the feed line, several diffraction peaks attributed to MoO_2_, MoO_3_, and NiMoO_4_ were detected at different two theta positions, whereas the Mo_2_C phases disappeared once the feed CH_4_/CO_2_ ratio was reached to 0.5/1 (Fig. 14d), suggesting that Mo_2_C could be readily oxidized by CO_2_ and then converted into an inactive MoO_2_ phase and CO. Adrianne et al*.*^72^ stated that MoO_2_ phases causing the gradual catalyst deactivation and the results clearly shown no coking after that. Nonetheless, in our study, it is quite worthy to note that even though the characteristic peaks of MoO_2_ phases are detected in the XRD pattern, the conversions exposed in Fig. 10 are remarkably constant and the catalyst seems not to deactivate at all. Moreover, MoO_2_ can be further oxidized into MoO_3_ with CO_2_ atmosphere_,_ this appears evident that MoO_3_ oxide phases have been widely distributed on the surface of the catalyst, which is in accordant with more micro-rods of molybdenum oxide observed in SEM analysis (Fig. 14d). Besides, it has been reported that the deposited carbon dynamically varied with MoO_x_ species because of its strong ability to allow the lattice oxygen (O^*^) to oxidize and remove the carbon formed during the DRM reaction^52^. This is also responsible for the absence of CNTs in this sample. Based on these results, we can conclude that the higher concentration of carbon dioxide in the entering gas, the less MWCNTs can be obtained.

It was also found that the main graphitic reflection peak at 2θ = 26.1° is predominant and even more dominant in the diffraction profile of the first sample test under a feed ratio of CH_4_/CO_2_ measured up to be 1.5 over the remaining peaks that reflected carbide, metallic, or other phases else. This can be indicated that the catalyst used under the condition mentioned earlier has superior performance to accumulate carbon nanomaterials and higher quality of deposited carbon can be achieved as illustrated in Fig. 14b.

**Figure 15:**
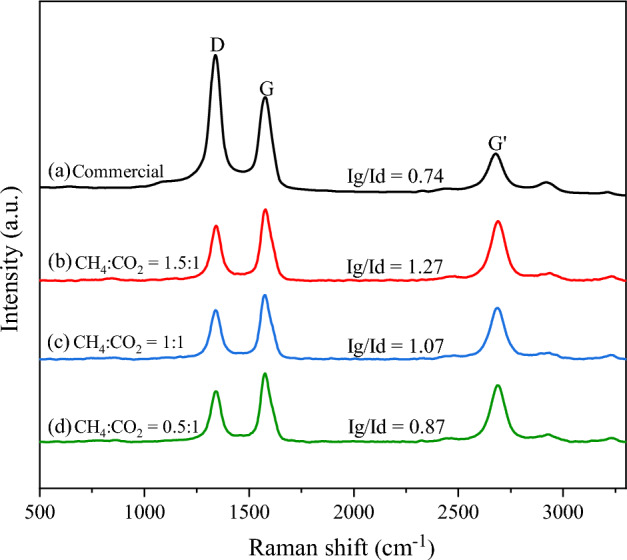
Raman spectra of (**a**) CNTs_commercial (Bayer®) and synthesized CNTs over NiMo/MgO catalyst at 800 °C with various feed gas ratio of CH_4_/CO_2_: (**b**) 1.5/1, (**c**) 1/1, and (**d**) 0.5/1.

The Raman spectra and the trend of I_g_/I_d_ ratio of synthesized CNTs obtained over NiMo/MgO catalyst at 800 °C using varied biogas ratios (CH_4_/CO_2_) of 1.5/1, 1/1, and 0.5/1 are shown in Fig. 15. The results present the I_g_/I_d_ intensity ratio of all synthesized CNTs (I_g_/I_d_ = 0.87–1.27) higher than commercial CNTs (I_g_/I_d_ = 0.74), which meant that the synthesized CNTs have a high quality. Noticeably, G-band's relative intensity to D-band (Ig/Id) is high in the CNTs synthesized using biogas at low CO_2_ content whereas it gradually decreases with the further increase of CO_2_ content in biogas. This tendency may principally be attributed to the oxidation effect of CO_2_ reacting with the graphitic layer in CNTs under operation at 800 °C^73^, leading to less structure quality of the CNTs.

### Durability of NiMo/MgO catalyst

**Figure 16:**
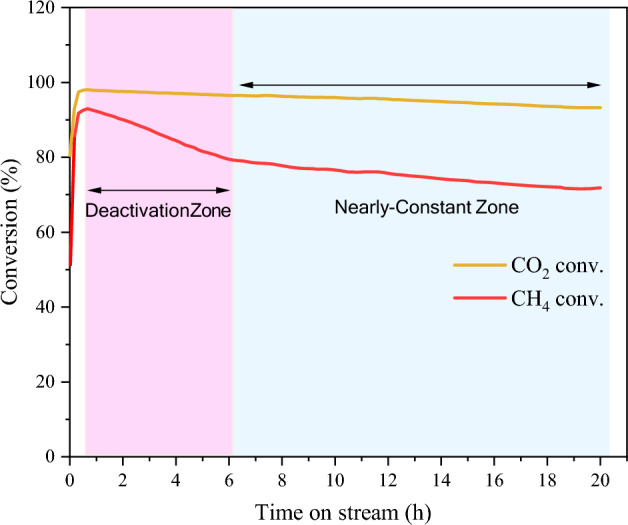
Catalytic activity expressed CH_4_ and CO_2_ conversion as a function of time on stream over NiMo/MgO catalyst during the reaction at 800 °C, 48,000 mL/g-h, 20 h.

Based on the previous results, the stability test of NiMo/MgO was carried out to probe the catalyst's longevity using a biogas ratio (CH_4_/CO_2_) of 1.5/1 over 20 h, as shown in Fig. 16. In Fig. 16, the catalyst was noticeably deactivated during the first 6 h of reaction with the methane and carbon dioxide conversion decreased from 92.98% and 98.04% to 79.27% and 96.51%, respectively. Afterward, the catalytic performance was stable with a slight deactivation comparable to a 7.44% and 3.27% loss in methane and carbon dioxide conversion. The point of view of this behavior can be attributed that the catalyst being initially exposed to the reactant gas resulting in CNTs growth. Over time, the numerous entangled CNTs structure network covered the catalyst surface, hindering the diffusion of methane and carbon dioxide toward the catalyst particles. This appearance asserted by methane and carbon dioxide greatly loss on the conversion as clearly perceived in the deactivation zone. After the reaction proceeds into the nearly constant zone, the CNTs production was continuously growing with a low formation rate compared to the deactivation zone due to a decreased surface of active metal attenuated methane and carbon dioxide decomposition rate. The CNTs production gradual growth may cause the catalytic performance to exhibit excellent stability. It can be speculated that it might exhibit stability with a reaction time of more than 20 h. This finding reinforces the notion that NiMo/MgO is an effective catalyst for biogas upgrading through the combination of dry reforming and catalytic breakdown of methane (CDM).

**Figure 17:**
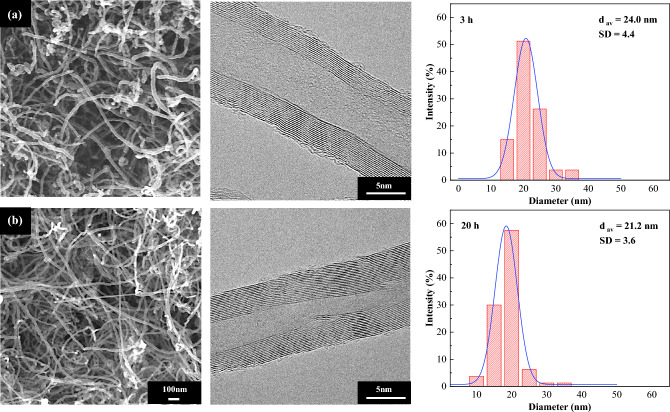
FE-SEM (× 50 k, 5 kV), HR-TEM (× 600 k, 300 kV) micrographs, and particle size distribution of CNTs grown over NiMo/MgO catalyst after running the reaction at 800 °C for (**a**) 3 h, and (**b**) 20 h.

Figure 17 presents FE-SEM, HR-TEM micrographs, and diameter distribution curves of carbon deposited over NiMo/MgO catalyst, which were later collected for characterization after the reaction at 800 °C with varied from 3 to 20 h time on stream. Apparently, all catalytic tests in this study produced a highly carbonaceous density of multi-walled CNTs. On top of that, it can be observed that some metal catalyst particle is embedded at the end of nanotubes as depicted in some white portion areas, which confirmed the formation of carbon nanotubes via the typical tip-growth mechanism^74,75^. These areas began to fade intensely away once reaction time was continually raised, demonstrating that the catalyst was being lifted off from the support material and elongated to form carbon nanofilament more and more, resulting in a high yield of solid carbon products. Moreover, no amorphous carbons were observed on the sidewall of tubular MWCNTs structure. This indicates that the produced CNTs in the 20 h time on stream test possess a greater degree of graphitization order. In addition, the length of MWCNTs seems to keep growing as much longer as time goes by.

The average diameter, the standard deviation, as well as the number of walls for the as-grown CNTs are calculated from SEM and TEM images, respectively, and be listed in Table 6. It is surprising to observe that an obvious decrease in the diameter nanotube was formed at a longer reaction time with a lower total inlet flow rate and amount of catalyst at half of usual that were applied for 3 h (200 ml/min and 0.25 g). The appropriate description for this finding is that the concentration of reactant gases per surface area is relatively low, and the catalyst layer was slightly thin. Thus, the carbon atoms were then distributed towards the catalyst particles in low magnitude. The carbon is easily diffused through the bottom layer. Consequently, large quantities of MWCNTs with small diameters can be obtained. On the other hand, at a higher feed flow rate with a very thick layer of the catalyst bed together, a huge number of carbon atoms are deposited intensely on the catalyst surface, leading to the aggregation of carbon species around metal particles, and therefore corresponds to enlarging of catalyst particle dimension and later holding somewhat larger diameter CNTs^76,77^.

**Table 6:** The result data evaluated from SEM, TEM images for CNTs synthesized after during different reaction time.

Sample	Catalyst weight (g)	Reactant flow rate (ml/min)	GHSV (ml/g-h)	d_av_^a^ (nm)	SD.^b^	No. of wall
CNTs_3h	0.5	400	48,000	24.0	4.4	11–34
CNTs_20h	0.25	200	48,000	21.2	3.6	12–21

^a^Diameter of CNTs calculated by mode.

^b^Standard deviation of CNTs diameter.

## Conclusion

This work has investigated the influence of process parameters on the catalytic performance of NiMo/MgO catalyst for converting biogas into syngas and carbon nanotubes. Multi-walled carbon nanotubes (MWCNTs) with the smallest diameter and the highest graphitization can be produced at 1000 °C. However, at higher temperature of 1100 °C, the agglomeration of metal particles due to the sintering was observed, resulting in the larger and broader diameter distribution of CNTs. Interestingly, the highest carbon yield and the uniform CNT diameter can be achieved at 800 °C as the optimum temperature, being a promising condition for mass production with low energy consumption. In addition, the catalyst can perform well with high stability more than 20 h at 800 °C. The effect of biogas composition was examined. The typical biogas composition of CH_4_/CO_2_ of 1.5 showed positive results in terms of enhancing production yields of CNT and syngas with negligible drop in the catalyst activity. Although the high fraction of CO_2_ in biogas caused the formation of MoO_3_ species, the catalyst activity was unchanged. The combination of Ni and Mo shows the synergistic property and suitable composition for utilizing in the biogas conversion at the various temperatures and gas compositions.

## Data Availability

All data related to the finding of this study are accessible upon request from the corresponding author Sakhon Ratchahat.
